# Viewing Inflammation and Immunoregulation Under the Calpain System Lens

**DOI:** 10.3390/cells14221814

**Published:** 2025-11-19

**Authors:** Vijay Kumar, John H. Stewart

**Affiliations:** Laboratory of Tumor Immunology and Immunotherapy, Department of Surgery, Morehouse School of Medicine, 720 Westview Drive, Medical Education Building-C, Atlanta, GA 30310, USA

**Keywords:** calpains, inflammation, immunoregulation, adaptive immunity, innate immunity, immune cells

## Abstract

The controlled pro-inflammatory immune response is critical for fighting against external and endogenous threats, such as microbes/pathogens, allergens, xenobiotics, various antigens, and dying host cells and their mediators (DNA, RNA, and nuclear proteins) released into the circulation and cytosol (PAMPs, MAMPs, and DAMPs). Several pattern recognition receptors (PRRs) and their downstream adaptor molecules, expressed by innate and adaptive immune cells, are critical in generating the inflammatory immune response by recognizing PAMPs, MAMPs, and DAMPs. However, their dysregulation may predispose the host to develop inflammation-associated organ damage, neurodegeneration, autoimmunity, cancer, and even death due to the absence of the inflammation resolution phase. The cytosolic calcium (Ca^2+^) level regulates the survival, proliferation, and immunological functions of immune cells. Cysteine-rich proteases, specifically calpains, are Ca^2+^-dependent proteases that become activated during inflammatory conditions, playing a critical role in the inflammatory process and associated organ damage. Therefore, this article discusses the expression and function of calpain-1 and calpain-2 (ubiquitous calpains) in various innate (epithelial, endothelial, dendritic, mast, and NK cells, as well as macrophages) and adaptive (T and B cells) immune cells, affecting inflammation and immune regulation. As inflammatory diseases are on the rise due to several factors, such as environment, lifestyle, and an aging population, we must not just investigate but strive for a deeper understanding of the inflammation and immunoregulation under the calpain system (calpain-1 and calpain-2 and their endogenous negative regulator calpastatin) lens, which is ubiquitous and senses cytosolic Ca^2+^ changes to impact immune response.

## 1. Introduction

Inflammation was known to ancient Ayurvedic physicians of the Indian peninsula, dating back to 1500 BCE and 600 CE, as depicted in their ancient Sanskrit-language textbooks as elevation, edema, heaviness, and pain [1,2,3]. Thereafter, Greek physicians, such as Hippocrates (5th century BCE), introduced the term edema for inflammation, and Aulus Celsus (30 BCE-38 CE) introduced four cardinal signs of inflammation: *Rubor* (redness), *Calor* (Heat), *Tumor* (swelling), and *Dolor* (pain) [1,2]. Later on, Galen (129-210 AD) and Virchow (1871) added and detailed the fifth sign of inflammation, namely, loss of function (*function lasea*) of the affected tissue or organ, viewing inflammation as inherently pathogenic or pathologic to the host [4,5,6]. Over time, advances in the medical and biomedical sciences, especially in immunology, have increased our understanding of the inflammatory process. For example, immunology explained the critical involvement of immune cells and their factors (cytokines, chemokines, and the complement system) in the process of inflammation. Moreover, we now know that immunological well-being, controlling an individual’s homeostasis via maintaining the optimum immune function, is critically needed to fight against endogenous (dead cells and their extracellular components, such as DNA, mRNA, and proteins) and exogenous threats (pathogens, allergens, carcinogens, and traumatic injuries), called immune homeostasis or immunohomeostasis [7].

For example, the local tissue immune microenvironment comprises residential immune cells, such as tissue-resident macrophages, dendritic cells (DCs), Langerhans’s cells (LCs), innate lymphoid cells (ILCs), and different types of T cells, including tissue-resident memory T cells (TRMs), which serve as guardians of organ homeostasis by recognizing and clearing foreign and endogenous threats [8,9,10]. Therefore, we now define inflammation broadly as a protective immune mechanism that fights external or endogenous threats and resolves upon threat clearance. However, in some cases, the inflammatory process does not resolve. Unresolved inflammation or a dysregulated inflammatory process is detrimental to the host, serving as a critical factor in several chronic inflammatory diseases, such as allergies, autoimmunity, auto-inflammatory diseases, cancers, metabolic syndrome, and neurodegenerative diseases [6,11]. The inflammatory immune response involves the recognition of potential threats [pathogen-associated molecular patterns (PAMPs) and/or microbe-associated molecular patterns (MAMPs), allergens, foreign antigens, xenobiotics, and endogenously induced damage/death-associated molecules (DAMPs)] by corresponding receptors, like pattern-recognition receptors (PRRs) and downstream signaling events that are critical for the synthesis and release of several pro-inflammatory immune mediators, such as cytokines, chemokines, interferons (IFNs), and reactive oxygen and nitrogen species (ROS and RNS) [1,12]. This process also involves several proteases, such as the calpain system, and their roles in inflammatory and immunoregulatory processes are understudied [13,14].

The calpain system comprises two calcium (Ca^2+^)-dependent cysteine-rich ubiquitously expressed non-lysosomal cytosolic proteases, called calpain-1 (μ-calpain) and calpain-2 (m-calpain), and one calpastatin (CAST) polypeptide, which is an endogenous negative regulator of calpains [15,16]. Calpain-1 is referred to as μ-calpain because it requires a micromolar Ca^2+^ concentration for activation, whereas calpain-2 depends on a millimolar Ca^2+^ level; therefore, it is also referred to as m-calpain. The calpain–calpastatin interaction that negatively regulates two calpains has been discussed in detail elsewhere [15,17]. Calpains play a crucial role in cytosolic Ca^2+^-dependent cellular functions, including cytoskeletal remodeling, cell cycle progression, gene expression, and cell death processes, such as apoptosis and necroptosis, which are critical events in the pathogenesis of inflammation and immunoregulation [18,19,20,21]. In addition, calpain-mediated cleavage of cytoskeletal vimentin (a type III intermediate filament protein) during acute inflammatory conditions and infections in immune cells, such as macrophages, induces their pyroptosis (an inflammatory cell death derived by membrane pore formation, inducing cell rupture due to osmotic influx), which further releases macro-DAMPs, such as mitochondrial DNA and the mitochondrial cytochrome c oxidase 1 or Cox1 to aggravate the inflammatory process due to their immunostimulatory nature [22]. Hence, understanding inflammation and immunoregulation is critical, particularly through the lens of the calpain system, which can be utilized to target inflammatory diseases through immune cell-specific calpain targeting. Therefore, the current article discusses the immunoregulatory and inflammatory effects of calpains, focusing on their expression and functions in primary immune cells, which are crucial for maintaining immune homeostasis.

## 2. Calpain (Calpain-1 and -2) Expression and Actions in Different Immune (Innate and Adaptive) Cells

### 2.1. Epithelial Cells

Epithelial cells are now considered potent innate immune cells due to expression of different PRRs, such as toll-like receptors (TLRs), nucleotide-oligomerization domain (NOD)-like receptors (NLRs), RIG-1-like receptors (RLRs), and melanoma-differentiation-associated gene-5 (MDA-5), and they produce different innate immune mediators critical for antimicrobial, inflammatory, and immunoregulatory functions [23,24,25,26,27,28,29,30,31]. They even interact with innate and adaptive immune cells to maintain immune homeostasis and also release several mediators, such as thymic stromal lymphopoietin (TSLP), IL-33, and B cell activating factor (BAFF), which modulate local accumulation and activation of Th2 responses and B cell immunoglobulin (Ig) production during different allergic and chronic inflammatory diseases [23,32,33,34]. Moreover, epithelial cells, such as nasal epithelial cells and primary airway epithelial cells (AECs), also generate types 1 (IFN-α and -β) and III (IFN-λ) interferons (IFNs) in response to microbial infections, such as severe acute respiratory syndrome-Coronavirus-2 (SARS-CoV-2) and influenza A viruses (IAVs) [24,35]. TLR3 activation is critically involved in the production of types I and III IFNs by primary AECs via signal transducer and activator of transcription 1 (STAT1) activation [24]. At the same time, activation of pulmonary epithelial cells during *Mycobacterium tuberculosis*-induced airway infection/tuberculosis generates type II IFNs (IFN-γ) in a STAT1 activation-dependent manner to exert nitric oxide (NO^.^)-dependent mycobactericidal effects [36].

Additionally, AECs also express and secrete various complement proteins, including complement component 3 (C3), C5, and factor B, which are crucial for their complement-associated immunoregulatory effects and survival during acute infections or sterile inflammation-associated insults [37,38,39,40]. For example, endogenous C3 of AECs and their tendency to load exogenous C3H_2_O rescues them from cell death induced by several factors, such as hydrogen peroxide (H_2_O_2_) and growth factor deprivation [37]. However, C3 mitigates AEC death in some conditions, independent of apoptosis and pyroptosis [37]. These AECs secrete C3 constitutively, which further increases during infectious or inflammatory conditions. For example, AEC C3 protects against pneumonia-associated acute lung injury (ALI), a condition that also requires the alternative complement component factor B [41]. Thus, skin epithelial cells and epithelial cells at mucosal surfaces are potent innate immune cells that create a borderline defense between the host and the external environment, which further signals adjacent or local and distant immune cells via direct interaction or the secretion of immune mediators.

**Figure 1:**
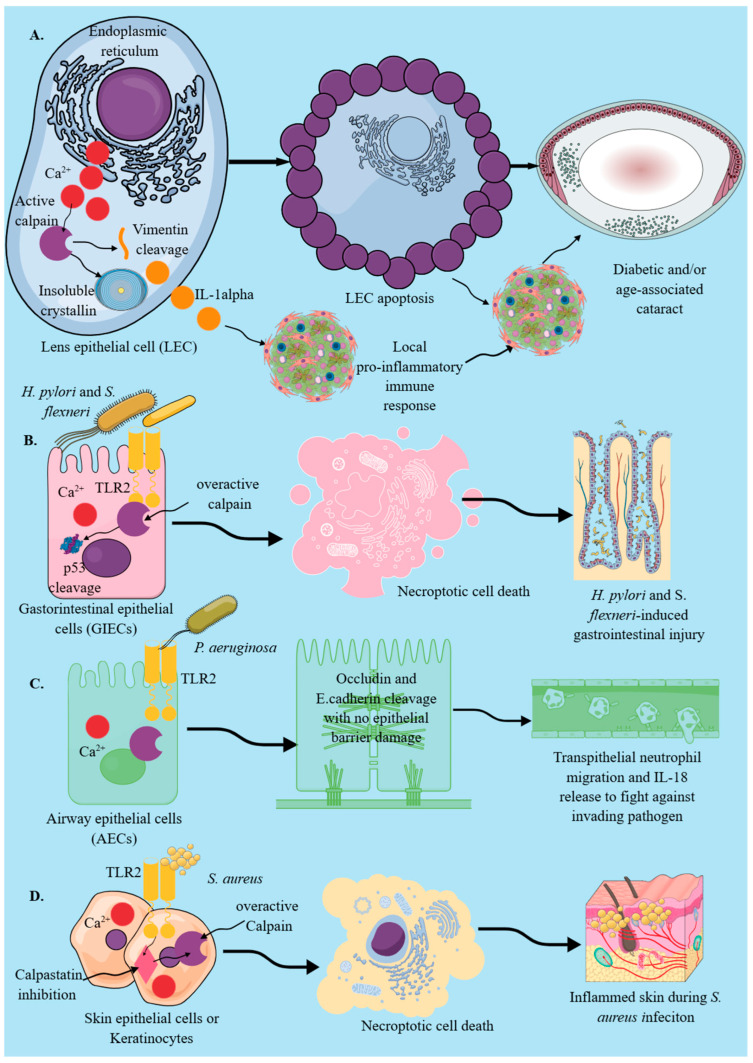
Calpains in different epithelial cells and their role in their pro-inflammatory action. (**A**). Calpain activation in LECs during diabetic or age-associated cataract induces cytosolic Ca^2+^-dependent calpain activation, which cleaves cytoskeletal vimentin protein and induces insoluble crystallin and mature IL-1α release, inducing local pro-inflammatory immune response and LEC apoptosis. (**B**). During infections caused by *H. pylori* and *S. flexneri*, gastrointestinal epithelial cells induce necroptosis via TLR2-dependent calpain activation, which cleaves p53 to prevent their apoptosis. This causes *H. pylori* and *S. flexneri*-induced gastric ulcers and intestinal injury. (**C**). During *P. aeruginosa* lung infection, AECs induce transepithelial migration of neutrophils to contain the infection without damaging the epithelial barrier in a calpain-activation-dependent manner via the TLR2 signaling pathway. (**D**). During *S. aureus* infection, skin epithelial cells or keratinocytes activate calpains via TLR2 signaling-dependent cytosolic Ca^2+^ influx, which induces their necroptosis, aggravating infection and associated inflammation.

Calpain-1 (*Capn1)* and calpain-2 (*Capn2*) are expressed in different epithelial cells, such as respiratory, gastrointestinal, urinary tract, and reproductive tract cells, including the breast tissue environment, and human lens epithelial cells (LECs), where they play a critical role in epithelial cell immune-modulatory functions and death under different inflammatory insults, including infectious diseases, ischemia, and cataract (Figure 1) [15,42,43,44]. Moreover, a significant increase in calpain (calpain-1 and calpain-2) expression and activity due to an increased release of Ca^2+^ from endoplasmic reticulum (ER) in LECs of patients with diabetic cataract has been observed, which is directly associated with apoptotic death of LECs via their structural proteins, such as vimentin, supporting transformation of soluble crystallin into insoluble truncated form, which scatters light (Figure 1A) [45,46,47,48]. Recent data has suggested the involvement of the immune system in cataract pathogenesis, as the calpastatin level in human LECs decreases with aging, causing overactivation of calpains, which might induce the release of pro-inflammatory cytokines, such as IL-1α, and acquire the senescence-associated secretory phenotype (SASP), activating local innate immune cells and recruiting distant immune cells to create a tissue-destructive pro-inflammatory environment [43,49]. Moreover, calpain activation induces their death, which further stimulates the pro-inflammatory immune response. Thus, calpain activation in LECs within the immune-privileged environment of the lens triggers a pro-inflammatory response, potentially aggravating or initiating the pro-inflammatory events associated with age-related cataract, which has a significant correlation with the activation of pro-inflammatory immune responses (Figure 1A).

#### Signaling Events That Stimulate Calpain Activation in Epithelial Cells to Induce Their Immunological Functions

TLRs are critical PRRs that regulate innate and adaptive immune responses during various infectious and inflammatory conditions to maintain immune homeostasis [7,50,51,52,53,54]. Epithelial cells at various mucosal surfaces express different TLRs, and the interaction between gut microbiota and gut epithelial TLRs is critical for maintaining gut homeostasis, including gut immune homeostasis [55,56,57]. The loss of this equilibrated gut epithelial TLR–microbiota interaction may cause inefficient clearance of pathobionts, potentially increasing the incidence of gastric infections due to disturbed immune homeostasis and microbiota. Critical illness, such as sepsis, may also cause gut failure, leading to a disturbed gut epithelial TLR–microbiota interaction, which may exert a long-lasting impact on local (gut) and systemic immunity [58]. Moreover, this prolonged alteration in the gut microbiota may further disrupt this homeostatic interaction, increasing the chances of developing colitis, inflammatory bowel disease (IBD), and colorectal cancer (CRC) [55]. Similar to the gut epithelial–microbiota interaction via TLRs, pulmonary epithelial cells (PECs), such as those in the nasal, tracheal, and bronchial epithelia, which express different TLRs, interact with their local microbiota to maintain local and systemic immune homeostasis [24,59,60,61]. Nasal epithelial cells and primary AECs highly express TLR3, 7, and 9, which enable them to sense pathogenic viruses and mount an initial innate immune response to maintain local immune homeostasis [60]. However, an altered lung microbiome, like an altered gut microbiome, is also associated with chronic pulmonary inflammatory diseases, such as allergic asthma, chronic obstructive pulmonary disease (COPD), frequent episodes of pneumonia, and even lung cancer [62]. Skin epithelial cells, such as keratinocytes, also express various TLRs, which interact with the local microbiota to maintain a healthy skin microbiome, homeostasis, and immune homeostasis [63,64,65]. Altered skin microbiota is associated with various chronic inflammatory skin diseases, including acne vulgaris and atopic dermatitis (AD) [66]. Thus, epithelial cell TLRs play a critical role in maintaining a healthy local microbiota (gut, lungs, and skin) and immune homeostasis.

The activity of cytosolic calpains depends on intracellular Ca^2+^ levels. TLR signaling has been shown to increase cytosolic Ca^2+^ levels by interacting with Ca^2+^ sensing proteins, such as STIM1 (stromal interaction molecule 1), which controls Ca^2+^ flow into the cell by activating different Ca^2+^-release-activated channels (CRACs), such as Orai1 [67,68,69]. Knockdown of these CRACs or chelation of cytosolic Ca^2+^ decreases the TLR (TLR3 and TLR4) stimulation-induced TNF-α and IL-6 release by innate immune cells, such as astrocytes, by decreasing cytosolic Ca^2+^ levels by regulating store-operated Ca^2+^ entry (SOCE) pathways [68,69]. LPS-stimulated CD14-dependent intracellular Ca^2+^ fluxes also induce TLR4 internalization/endocytosis, a critical step for downstream TRIF-related adaptor molecule (TRAM) and Toll/IL-1 receptor (TIR) domain-containing adaptor-inducing IFN-β (TRIF)-dependent signaling, which promotes the expression of interferons (IFNs) and IFN signaling genes (ISGs) [70,71,72]. Endosomal TLR4, which contains a TIR domain, interacts with phospholipase Cγ2 (PLCγ2), spleen tyrosine kinase (SYK), and E1/E2 ubiquitination and undergoes degradation as it lacks canonical TLR signaling adaptor molecules and cascades [73]. Endosomal TLR2 signaling via TRAM is also critical for the type 1 IFN response during herpes simplex virus (HSV) and *Staphylococcus aureus* (*S. aureus*) infection, facilitating the clearance of the infection [74]. Moreover, TLR9 signaling is also significantly attenuated (decreased IL-1α and IL-1β secretion) in the presence of Ca^2+^ chelators (EGTA-AM) and calcineurin inhibitor (FK-506) due to the inhibition of IκBβ (an NF-κB inhibitory protein) degradation [75]. Thus, TLR signaling increases cytosolic Ca^2+^ levels, which are crucial for maintaining normal immune homeostasis during host–microbiota interactions in various compartments and in response to infectious and inflammatory conditions, thereby enabling the defense against diverse pathogens and inflammatory agents.

TLR2 signaling in different epithelial cells, such as gastric, intestinal, skin, and urinary tract epithelial cells, activates calpain activity in response to different pathogens, such as *Helicobacter pylori* (*H. pylori*), *Staphylococcus aureus* (*S. aureus*), *Chlamydia trachomatosis* (*C. trachomatosis*), and *Neisseria gonorrhoeae* (*N. gonorrhoeae*) (Figure 1B–D) [76,77]. The activation of calpains in human gastric epithelium in response to chronic TLR2 stimulation promotes inflammatory tissue damage and disrupts adherens junctions, as seen during *H. pylori* infection, which is a critical risk factor for gastritis, gastroduodenal ulcers, and gastric adenocarcinoma induction and development (Figure 1B) [76]. Moreover, patients with *H. pylori* infection exhibit elevated circulating 80-kDa E-cadherin ectodomain. TLR2 inhibition not only blocks calpain activity but also adherent junction disassembly. Similarly, *Shigella flexneri* (*S. flexneri*) infection, via TLR4 and TLR2 activation in intestinal epithelial cells (IECs), activates calpains by increasing cytosolic Ca^2+^ (Figure 1B) as a result of genotoxic stress induction and its virulence factor, VirA [77,78].

Activated calpains cleave p53 (critical for DNA repair) to prevent its apoptotic death, thereby supporting the pathogen’s growth and multiplication inside infected IECs, which later induces necroptosis and exaggerated inflammatory tissue damage (Figure 1B) [77,78]. Moreover, IEC Shigella infection alters the sumoylation process by activating calpains, which inhibit the SUMO E1 enzyme SAE2, thereby supporting its entry into IECs by limiting cytoskeletal rearrangements induced by bacterial effectors [79]. Sumoylation plays a critical role in host defense during *S. flexneri* infection by regulating intestinal permeability and restricting epithelial invasion, thereby controlling mucosal inflammation [80,81]. Thus, IEC calpain activation during *S. flexneri* infection not only expedites the IEC infection process by modifying the cellular architecture through the cleavage of different cell membrane proteins but also prevents their apoptosis via p53, supporting their intracellular growth and multiplication (Figure 1B). However, TLR (TLR2 and TLR4)-dependent local macrophage activation during shigellosis produces IL-12, which induces IFN-γ production from local innate lymphocytes (NK and γδT cells) and controls *S. flexneri* replication in IECs [82,83]. IECs can also produce IFN-γ, which may protect intestinal epithelial barrier integrity by inducing the production of IL-18 binding protein (IL-18bp), comprising the IL-18/IL-18bp system (an anti-inflammatory system) and inducing their early apoptosis to prevent excess pathogen growth and inflammatory tissue damage [84,85]. Thus, it will be interesting to observe the impact of calpain activation on IFN-γ production during epithelial cell infections, such as those caused by *S. flexneri*. The selective protein targets of calpains, critical for cell motility and adherence, have been discussed in detail elsewhere [18,19,86].

On the other hand, in the pulmonary lung microenvironment, TLR2 stimulation-mediated calpain activation in PECs during *Pseudomonas aeruginosa* (*P. aeruginosa*) infection cleaves occludin and E-cadherin (transmembrane junctional proteins) without disturbing epithelial barrier integrity (Figure 1C) [87,88]. The calpain-mediated cleavage of occludin and E-cadherin is critical for the transepithelial migration of neutrophils to combat invading respiratory pathogens, along with the release of chemokines, such as IL-8 (Figure 1C). Hence, TLR2 signaling-induced cytosolic Ca^2+^ upregulation activates calpains in mucosal epithelial cells, which facilitates the transepithelial migration of potent innate immune cells, such as neutrophils, to combat invading pathogens and control mucosal inflammation by regulating epithelial barrier functions (Figure 1C) [88,89]. Moreover, in calpain-1 and calpain-2 conditional knockout mice subjected to acute bacterial peritonitis, decreased neutrophil infiltration and associated bacterial clearance have been observed, indicating a critical role of calpains in neutrophil infiltration at the site of infection [90]. The peritoneum is a simple squamous cell epithelial lining of the abdominal cavity. These studies further indicate that the activation of calpain in epithelial cells and myeloid innate immune cells (MICs) during bacterial infections is critical for clearing the infection and maintaining immune homeostasis.

The signal strength and duration of TLR signaling in epithelial cells are critical determinants of calpain-mediated tissue-damaging effects, such as epithelial barrier damage resulting from cell death, as observed during *H. pylori* infection. Moreover, during *S. aureus* skin infection, keratinocytes die due to their necroptosis in response to overactivated calpains in response to TLR2 signaling and calpastatin (a natural calpain inhibitor) inhibition (Figure 1D) [77,91]. Furthermore, calpain activity is critical for clearing the infection during acute bacterial peritonitis, and its deficiency leads to the development of bacteremia that can progress into sepsis if the infection has not been cleared [90]. Hence, calpains are critical for epithelial cell-mediated immunoregulatory functions and inflammatory processes.

### 2.2. Endothelial Cells

**Figure 2:**
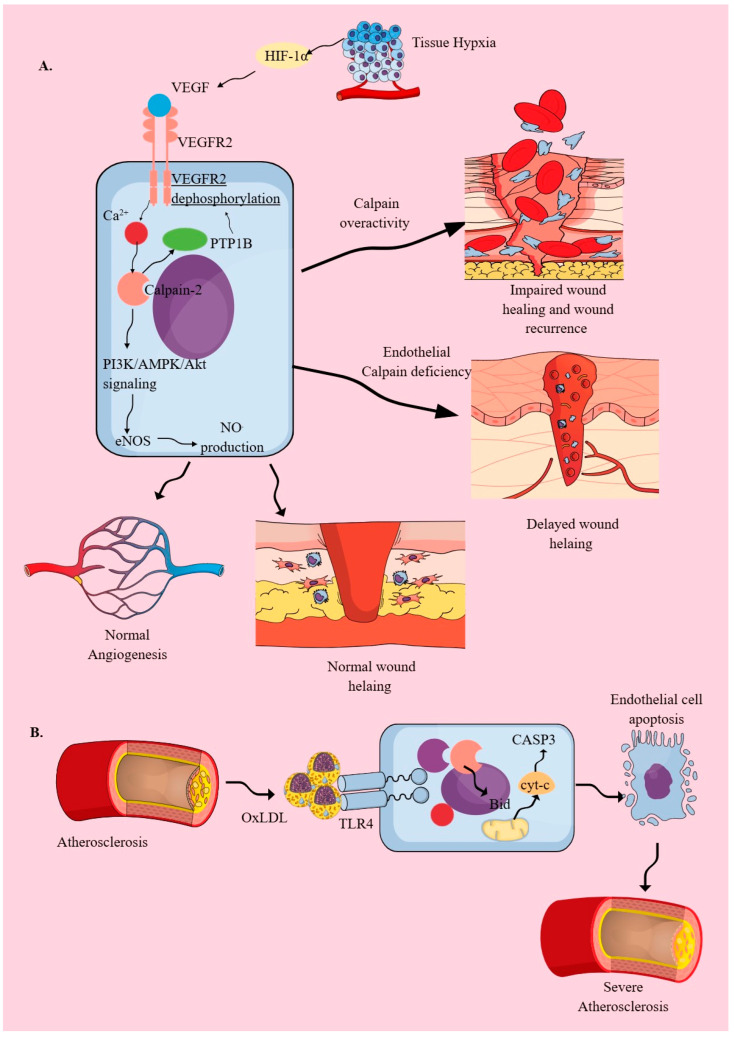
Endothelial calpains and their physiological and immunological functions. (**A**). Under normal conditions, in response to VEGF via VEGFR2 interaction, endothelial calpains induce calpain activity, which, via PI3K/AMPK/Akt signaling, induces eNOS activation. Activated eNOS generates NO^.^, which is critical for angiogenesis and wound healing. However, overactivated calpain activity in diabetic patients impairs the wound healing process, and even healed wounds recur. Moreover, endothelial cell calpain deficiency delays wound healing. (**B**). In atherosclerosis, oxLDLs activate TLR4, which, via calpain activation, cleaves Bid and induces cytochrome c (cyt-c) release from the mitochondria. Cyt-c induces CASP3 activation and endothelial cell apoptosis, which increases atherosclerosis severity.

Vascular endothelial cells exhibit structural and barrier function heterogeneity depending on the organ studied, as discussed in detail elsewhere [92,93]. In addition to their vascular and barrier functions, endothelial cells are also considered as innate immune cells due to expression of different active PRRs, antigen presentation, phagocytosis, and secretion of several inflammation mediators, such as cytokines, chemokines, and type 1 IFNs [51,94,95,96,97,98,99]. Therefore, due to their role as innate immune cells and because they line blood vessels, endothelial cells critically orchestrate the inflammatory process, such as immune cell trafficking and release of pro-inflammatory mediators [100,101]. Endothelial cells also express calpains, which are crucial for their angiogenic function and inflammatory response upon exposure to inflammatory stimuli, inducing changes in their expression and activity that regulate transcellular permeability (Figure 2A) [102,103]. For example, vascular endothelial growth factor-2 (VEGF-2) activates calpain-2 (m-calpain) to promote angiogenesis under normal conditions without involving calpain-1 or μ-calpain by activating phosphatidylinositol 3 kinase (PI3K)/5′-Adenosine monophosphate (AMP)-activated protein kinase (AMPK)/Akt-dependent endothelial nitric oxide synthase (eNOS) phosphorylation and nitric oxide (NO^.^) production in endothelial cells (Figure 2A) [102,104,105,106]. This VEGF-dependent angiogenic effect on endothelial cells is regulated by a calpain-dependent negative feedback loop that inhibits overactivation of VEGF receptor 2 (VEGFR2) (Figure 2A) [107]. Calpain cleaves and activates protein tyrosine phosphatase type 1B (PTP1B), which dephosphorylates VEGFR2 (Figure 2A) [107].

Failure of this calpain-dependent negative feedback (PTP1B inhibition or overexpressed/overactive calpains) is seen in impaired wound healing in experimental animals with diabetes that develop diabetic wounds (Figure 2A) [107]. Moreover, diabetes-associated high glucose concentrations, or hyperglycemia, downregulate focal adhesion kinase (FAK) expression by inducing calpain-1 overexpression, which cleaves FAK explicitly and is responsible for the abnormal architecture of healed diabetic ulcers and recurrence [108]. However, under non-diabetic conditions, the targeted deletion of endothelial calpains (CAPNS1, the common regulatory subunit of calpain-1 and calpain-2) decreases the wound healing process by reducing their inflammatory functions, such as the generation of NF-κB-dependent pro-inflammatory cytokines (TNF-α, critical for endothelial cell proliferation, migration, and tube formation) and downregulating β-catenin expression (Figure 2A) [109]. Thus, in diabetic wounds, overactive calpains impair the wound-healing process, whereas calpain inhibition impairs the normal wound-healing process (Figure 2A).

IFN-induced protein 10 (IP-10) or CXC motif chemokine ligand 10 (CXCL10) via binding to CXCR3 inhibits VEGF-induced endothelial tube formation and motility by inhibiting calpain activity via upregulating cAMP and protein kinase A (PKA) activity [105]. CXCL10 levels have been found to increase in healing and non-infected diabetic wounds, which, by inhibiting calpains, may induce impaired wound healing [110]. In addition, CXCL10 is associated with the resolution of active proliferative diabetic retinopathy and the development of traction diabetic retinopathy, which correlates well with VEGF levels and activity on retinal endothelial cells [111]. Thus, it is interesting to study the CXCR3/CXCL10/calpain axis in the context of VEGF in patients with diabetic wounds and retinopathy, which lack proper healing and re-emerge after healing. On the other hand, lower circulating levels of CXCL10 are associated with diabetic kidney disease, characterized by excess fibrosis, which may be due to increased calpain activity [112]. Furthermore, calpain inhibition has shown beneficial effects in a swine model of myocardial fibrosis in chronic ischemic hypercholesterolemia [113]. Thus, understanding endothelial calpain regulation is a critical factor in combating various inflammatory and wound-healing issues associated with diabetes and other chronic inflammatory diseases, such as chronic artery disease.

VEGF also downregulates calpastatin (an endogenous calpain inhibitor that prevents the induction of pathogenic angiogenesis) expression in endothelial cells, which is observed during chronic inflammatory conditions, including cancers that exhibit altered angiogenesis or neoangiogenesis in response to pro-inflammatory cytokines, such as IL-6 and VEGF [114]. Endothelial calpastatin, by inhibiting calpains, prevents degradation of the suppressor of cytokine signaling 3 (SOCS3) molecule, keeping in check the IL-6/STAT3/VEGF-C axis-mediated neoangiogenesis in inflammatory hypoxic environments seen in the tumor microenvironment (TME), diabetic retinopathy, and non-healing wounds [114,115,116].

In addition to VEGF-1, fibroblast growth factor (FGF) and epidermal growth factor (EGF) also activate calpain activity in endothelial cells [102]. Hypoxia also elevates calpain expression and activity in endothelial cells, which inhibits the NF-κB inhibitory activity of IκB and promotes inflammatory events in these cells, a process essential for normal wound healing, as discussed earlier (Figure 2A) [109,117,118]. Furthermore, hypoxia via hypoxia-inducible factor-1α (HIF-1α) also elevates VEGF expression and Na^+^/H^+^ exchanger-1 (NHE1) expression, which further activates calpain expression and function under chronic hypoxic conditions (Figure 2A) [119]. Calpain-1 mediates HIF-1α expression during hypoxia through NF-κB (P65) activity [120].

Atherosclerosis is a chronic inflammatory condition that affects the vascular endothelium. Obesity, a high-fat diet (HFD), and cigarette/tobacco smoking are critical factors for atherosclerosis development. Patients with atherosclerosis or the aforementioned lifestyles have high circulating oxidized low-density lipoproteins (OxLDLs), which endothelial TLR4 recognizes to initiate an inflammatory cascade, including increases in intracellular Ca^2+^ and dependent calpain activity, as well as endothelial cell death and an increase in atherosclerosis severity (Figure 2B) [51,121,122,123,124,125,126]. OxLDL-induced endothelial cell death involves calpain-dependent Bid cleavage and subsequent cytochrome-C (cyt-c) release from the mitochondria, which activates caspase 3 (CASP3) (Figure 2B) [123]. OxLDL also induces pathogenic changes in the metabolism, transcriptome, and epigenome of endothelial cells before the induction of a typical inflammatory endothelial phenotype through AP-1, NFE-2, and CEBP transcription factors [127]. In addition to vascular endothelium, lymphatic endothelial cells also exhibit calpain dysregulation during hypercholesterolemia in response to lysophosphatidic acid, which limits their ability to stabilize regulatory T cells (T_regs_) and further supports inflammation [128]. The overexpressed and overactive calpain in lymphatic endothelial cells cleaves mitogen-activated protein kinase kinase kinase 1 (MEKK1) and subsequently cleaves its downstream target, TGF-β1. The inhibition of calpain in lymphatic endothelial cells inhibited inflammatory atherosclerotic plaque formation and increased T_regs_ in the peripheral circulation, which further reduced aortic atherosclerotic plaque formation in mice with hypercholesterolemia [128]. Moreover, lysophosphatidic acid-mediated overactive calpains increase the IL-18/NF-κB/vascular cell adhesion molecule-1 (VCAM-1) axis in lymphatic endothelial cells, thereby inhibiting lymphocyte mobility on the cells [128]. Calpain-1 deletion attenuates atherosclerotic plaque formation and improves vasomotor dysfunction in apolipoprotein E1 (ApoE1) knockout mice subjected to a high-fat diet (HFD) [129].

In genetically modified mice (endothelial-specific Capn4 knockout (TEK/Capn4^−/−^), LPS-induced acute endotoxemia did not develop severe acute kidney injury (AKI), as shown by wild-type (WT) mice [130]. Capn4 deletion in vascular endothelial cells protected them from AKI by preventing their apoptotic death, decreasing systemic and renal tissue reactive oxygen species (ROS) and NO^.^ levels/production due to the suppression of inducible and endothelial NOS (iNOS and eNOS) in kidney tissue [130]. The decreased iNOS activity was associated with lower calpain and associated p38MAPK activities in vascular endothelial cells. Moreover, traumatic brain injury (TBI)-induced hyperpermeability of the blood–brain barrier (BBB) also involves an overactive endothelial calpain system and can be reversed by inhibiting overactive calpain activity [131]. In addition to sterile inflammatory conditions, the endothelial cell infections seen during echovirus 1 (E1), Coxsackievirus B3 (CVB3), flaviviruses, and chikungunya virus activate calpain to support their entry, replication, endothelial cell polarization, and death, which may cause vascular leakage in severe cases, causing severe damage and patient death [77,132,133,134,135]. Thus, abrogated calpain activation in endothelial cells during severe inflammatory conditions, such as sepsis, induces their apoptotic death, breaches the endothelial barrier, and results in endothelial vascular leakage (increased inflammatory immune cell transendothelial migration, protein and fluid leakage), causing edema, organ damage, and death.

However, controlled vascular endothelial calpain activity is critical for diapedesis to fight against invading pathogens and inflammatory tissue insults, as calpain inhibition blocks transendothelial lymphocyte migration or diapedesis due to impaired development of intercellular adhesion molecule-1 (ICAM-1)-rich docking structures by endothelial cells [136,137,138]. Moreover, endothelial cells defective in myeloperoxidase (MPO) production show deficient calpain activity, eNOS production, and VCAM-1 expression, which is critical for diapedesis [137,139]. Hence, regulated calpain activity in endothelial cells is critical for the transendothelial migration of lymphocytes at the site of infection and inflammation, thereby maintaining homeostasis or immune homeostasis. However, endothelial cell overactivation of calpain may cause vascular leak, leading to exaggerated inflammation and organ damage, as seen in sepsis.

### 2.3. Calpains in Myeloid Innate Immune Cells (MICs)

MICs are generated in the bone marrow during hematopoiesis from common myeloid progenitors (CMPs), which are formed from multipotent progenitors (MPPs), generated from hematopoietic stem cells (HSCs), as discussed in detail elsewhere [140,141]. Moreover, hematopoiesis and myelopoiesis, which give rise to MICs, are further impacted by infections and other inflammatory processes [141,142,143,144]. Macrophages, neutrophils, dendritic cells (DCs), and mast cells are critical MICs with pro-inflammatory and immunoregulatory functions, which also regulate adaptive immunity through antigen presentation and their humoral factors (cytokines and chemokines) [1,145,146,147,148,149]. These MICs express several PRRs, recognizing endo- and exogenous threats, such as microbial infections and their products (MAMPs and PAMPs) and DAMPs, to serve as the first lines of immune defense [51,53,150,151,152]. All these MICs express conventional calpains (calpain-1 and calpain-2) and regulate their pro-inflammatory and immunoregulatory functions under homeostasis and different pathologies [153].

#### 2.3.1. Macrophages

**Figure 3:**
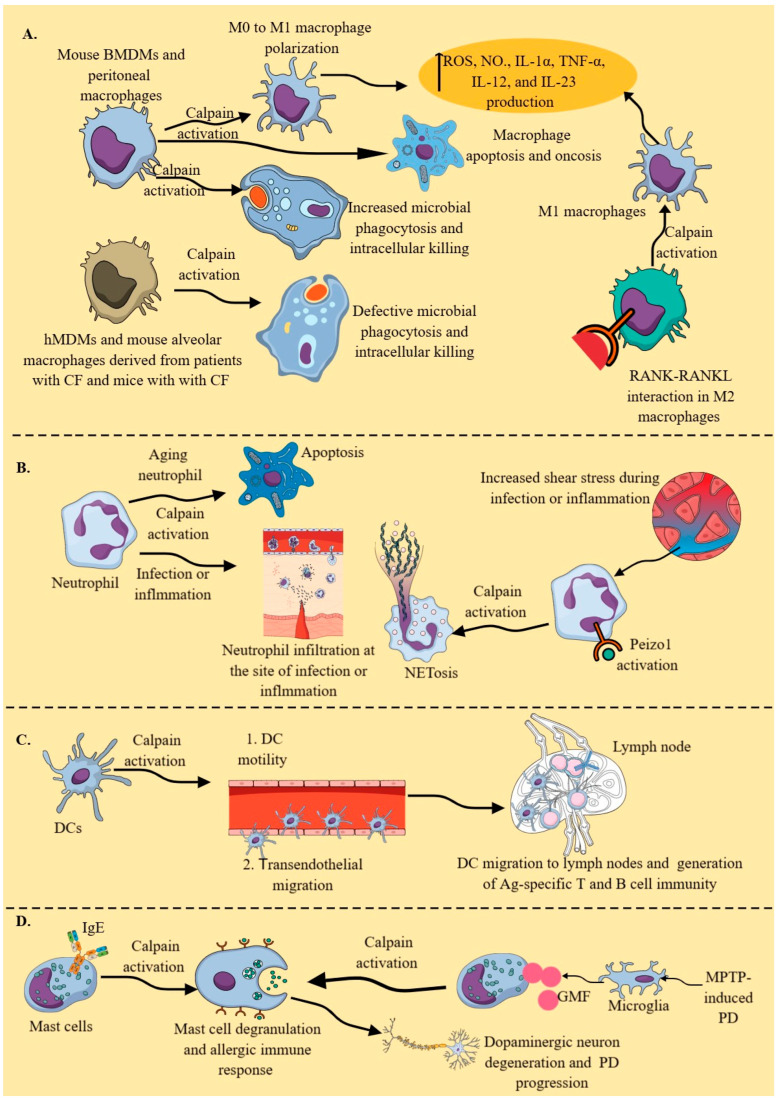
Calpains in MICs (macrophages, neutrophils, DCs, and mast cells). (**A**). In mouse BMDMs and peritoneal macrophages, during bacterial infections (please see the text), calpain activation is critical for M0 to M1 macrophage polarization, phagocytic killing of phagocytosed bacteria, macrophage apoptosis and oncosis, and release of several pro-inflammatory mediators. The RANK-RANKL interaction in M2 macrophages during infection and in the TME polarizes M2 macrophages to M1 macrophages via calpain activation. On the other hand, hMDMs and murine pulmonary macrophages isolated from mice and patients with CF calpain activation are associated with their defective phagocytic activity. (**B**). Under normal conditions, aging neutrophils overexpress calpains, which are critical for their apoptosis, but during infection or inflammation, neutrophil calpain activation is critical for their transendothelial migration/diapedesis. Moreover, neutrophil calpain activation in response to shear stress and piezo 1 activation induces NETosis, which is critical for inflammation and immune homeostasis maintenance. (**C**). Calpain activation in DCs is critical for their motility and transendothelial migration to reach lymph nodes during infection or in response to vaccination to generate Ag-specific T and B cell-dependent immunity. (**D**). Calpain activation in mast cells in response to IgE-mediated immune response induces mast cell degranulation and supports NF-κB-dependent pro-inflammatory action. Moreover, in an MPTP-induced mouse model of PD, microglia-derived GMF activates mast cells, which, via calpain activation, induces their degranulation and dopaminergic neuron degeneration, helping in PD progression. Kindly see the text for details.

During homeostasis or normal conditions, cytosolic calpains, which are mainly attached to endoplasmic reticulum (ER) membranes, specifically calpain-2, cleave selenoprotein K (SelK), which is also attached to ER membranes close to the operating calpain/calpastatin system [154]. Calpain-2 cleaves SelK into a truncated SelK form lacking a selenocysteine residue in resting or non-stimulated bone marrow-derived macrophages (BMDMs) derived from C57BL/6J mice. Additionally, another study indicated the presence of full-length SelK in *Capns1* KO naïve peritoneal macrophages of C57BL/6J mice [90]. However, in BMDMs stimulated with different TLRs, such as TLR2, TLR3, TLR4, and TLR9, increased calpastatin activity blocked the calpain protease activity responsible for cleaving SelK [154]. Full-length SelK is required for Ca^2+^ flux and macrophage migration at the site of inflammation in response to chemoattractants, such as monocyte chemoattractant protein-1 (MCP-1) [154]. Moreover, *Capns1* KO murine macrophages (including bone marrow-derived macrophages (BMDMs) and peritoneal macrophages) exhibit defective phagocytosis, characterized by decreased intracellular killing (ICK) of engulfed bacteria (Figure 3A) [90]. Decreased ICK may be associated with reduced intracellular reactive oxygen species (ROS) generation in peritoneal macrophages, as seen in *Capns1* KO neutrophils, which fail to kill enterobacteria in vivo in a mouse model of acute bacterial peritonitis [90].

In a pulmonary inflammatory environment, such as cystic fibrosis (CF), neutrophil elastase increases cytosolic Ca^2+^ and calpain-2 activation in murine alveolar macrophages and human monocyte-derived macrophages (hMDMs) from patients with CF, which impairs their phagocytic potential by cleaving cytoskeletal proteins (Talin and Ezrin) (Figure 3A) [155]. Moreover, neutrophil elastase also decreases calpastatin activity in murine alveolar macrophages isolated from mice with CF and hMDMs of patients with CF, which further increases calpain-2 activity. Interestingly, no differences in calpain activity, calpain-2 and calpastatin expression, and intracellular Ca^2+^ between CF and non-CF macrophages have been reported at baseline level. Thus, animal species, disease specificity, and the location of macrophages (organ specificity), along with external stimuli, play a critical role in calpain-mediated immune/inflammatory functions of macrophages, which must be studied [156,157,158,159]. For example, macrophages isolated from patients with CF exhibit abnormal expression and trafficking of TLR4, decreased expression of human leukocyte antigen (HLA)-DR and HLA-DQ (major histocompatibility complex-II (MHC-II) molecules that present antigens to CD4^+^T cells), and decreased phagocytic potential compared to those of normal humans [160,161,162,163].

Moreover, several other bacterial infections caused by *Streptococcus agalactiae* (a Group B streptococcus or GBS), *S. pyogenes* (a Group A streptococcus), *Bacillus anthracis*, *Listeria monocytogenes*, *Klebsiella pneumoniae*, and *Mycobacterium tuberculosis* utilize the calpain system for their survival, growth, and multiplication by inducing cytoskeletal derangements and decreasing phagosome and phagolysosome formation and apoptosis [77,164]. For example, *S. agalactiae* and *S. pyogenes* infections induce apoptosis and oncosis (a form of pro-inflammatory programmed cell death characterized by cell swelling and vacuolization of the cell cytoplasm, causing plasma membrane rupture) in murine peritoneal macrophages by increasing calpain activity (Figure 3A) [77,165,166,167]. The detailed mechanisms of macrophage apoptosis, including the role of calpains during GBS infection, have been discussed elsewhere [77,168]. Moreover, GBS activates the PI3K/AKT signaling pathway in THP-1 monocyte-like cells (ATCC TIB-202) treated with phorbol myristate (PMA) to behave as pro-inflammatory M1 macrophages, which also involves the calpain system for their intracellular survival [169]. Via listeriolysin O (LLO), *L. monocytogenes* in murine peritoneal macrophages (naïve and thioglycolate-treated mice) and J774 cells (murine macrophage cell line derived from BALB/c mice) utilizes the calpain system by increasing cytosolic Ca^2+^ levels to escape from phagosome maturation for their intracellular growth and multiplication [170,171]. Even *L. monocytogenes* utilizes calpain-2 to evade the intestinal immune barrier, such as Peyer’s patches (PPs), via iNOS-induced NO^.^, which, along with other critical immune cells, also includes macrophages [172,173,174]. Moreover, different *M. tuberculosis* strains also induce calpain overactivation in macrophages (RAW264.7 macrophages, alveolar macrophages, and BMDMs) by increasing cytosolic Ca^2+^ levels, which facilitates their apoptosis (Figure 3A) but prevents their autophagy, thereby allowing them to escape killing, as discussed in detail elsewhere [77,175,176,177,178]. Multidrug-resistant *K. pneumoniae* ST258 (KP35) inhibits Ca^2+^-dependent calpains in alveolar macrophages and neutrophils to avoid ICK in a murine pneumonia model [179]. Thus, calpains specific to macrophages are critical players in generating an antimicrobial immune response to clear the infection. Microbes have developed different strategies to hijack this system for their growth and multiplication.

In addition to different TLRs, receptor activator of NF-κB ligand (RANKL) also induces calpain overactivity in macrophages. For example, by activating calpain-1 activity, RANKL activation in RAW246.7 macrophages and BMDMs (mouse and rat) supports NF-κB activation and promotes their pro-inflammatory M1 macrophage phenotype, supporting osteoclastogenesis or bone formation [180,181]. Notably, RANKL-induced rat M1 macrophages (lower inducible nitric oxide (NO^.^) synthase (iNOS) expression), which support osteoclastogenesis, differ from M1 macrophages generated in the presence of LPS and INF-γ (higher iNOS expression) [181]. It will be interesting to explore these rat BMDM findings in murine and human macrophages.

Moreover, macrophage-specific RANK-RANKL interactions in immunosuppressive TIME of different cancers may convert a cold tumor to a hot (immune-responsive) tumor by converting immunosuppressive M2 macrophages (that express RANK) into pro-inflammatory/antitumor M1 macrophages (Figure 3A) [182]. Macrophage-specific RANK-RANKL interaction/stimulation has also been observed to polarize murine peritoneal M2 macrophages into M1 macrophages during *Leishmania major* infection, facilitating the clearance of the pathogen by generating ROS and NO^.^ (Figure 3A) [183]. Moreover, RANKL and IFN-γ exert a synergistic effect to generate M1 peritoneal macrophages, as indicated by the overexpression of iNOS and NO^.^ production to fight against *L. major* infection [183]. This further supports the notion that macrophage calpains are crucial in clearing infections by enhancing their antimicrobial and pro-inflammatory activities. The deletion of calpain small subunit 1 (*Capns1,* critical for stabilization and function of calpain-1 and calpain-2) prevents the polarization of M0 macrophages to pro-inflammatory M1 macrophages, which are critical for generating a pro-inflammatory immune response (Figure 3A) [184]. Calpain activation in macrophages regulates NF-κB- and PI3K/AKT1-dependent pro-inflammatory immune responses, such as ROS, NO^.^, IL-1α, TNF-α, IL-12, and IL-23 generation, which are critical for clearing infections and inflammatory disease pathogenesis (Figure 3A).

Nicotinic acetylcholine receptor alpha 1 (nAChRα1) stimulation in murine renal macrophages also activates calpain-1 and calpain-2 activities to induce their pro-inflammatory phenotype and function, as observed in the experimental mouse model of chronic hypercholesterolemic nephropathy [185]. The silencing of nAChRα1 significantly reduces both calpain-1 and calpain-2 activities and talin (a calpain substrate) degradation in murine renal macrophages isolated from an apolipoprotein E knockout (ApoE^-/-^) mouse model of chronic kidney disease. Interestingly, nAChRα1 is not highly expressed in other macrophage types. However, α7nAchRs are highly expressed on macrophages and other innate immune cells, where their stimulation with acetylcholine (Ach) and nicotine, two endogenous and exogenous ligands, suppresses the pro-inflammatory immune response in diverse inflammatory conditions, including pain and sepsis [186,187,188]. α7nAchR activation-mediated anti-inflammatory effects involve calpain inhibition, as it decreases cytosolic Ca^2+^ levels in alveolar macrophages activated by extracellular adenosine triphosphate (ATP) [189]. Extracellular ATP functions as a danger signal and potent inflammatory mediator by acting on extracellular P2X and P2Y purine receptors, including P2X7, which is responsible for generating the local and systemic inflammation that leads to organ damage, as seen during sepsis [190,191,192]. The ATP-P2X7 interaction on macrophages (hMDMs, murine BMDMs, and THP-1 cells) also induces calpain activation due to cytosolic Ca^2+^ increase, inducing unconventional protein secretion, followed by their necrosis and NLRP3 inflammasome activation, inducing IL-1β secretion [193]. Moreover, this ATP-induced NLRP3 activation and IL-1β secretion involves a calpain-dependent vesicle-mediated secretion pathway. Moreover, transgenic mice overexpressing calpastatin or CAST (natural negative regulators of calpains) subjected to an anterior coronary artery ligation-induced myocardial infarction (MI) exhibit higher mortality at six weeks than wild-type (WT) mice with a high incidence of cardiac rupture during the first week post-MI [194]. This is due to decreased infiltration of CD4^+^T cells (CD8^+^T cell infiltration remains unchanged) and increased infiltration of monocyte/macrophages, which are defective in undergoing anti-inflammatory M2 macrophage proliferation and exerting their wound-healing action [194]. However, it is essential to note that calpastatin overexpression and calpain knockout may employ distinct mechanisms to exert their immunoregulatory effects under various inflammatory conditions.

Obesity, which has become a pandemic, is immunologically characterized as a condition of chronic low-grade systemic inflammation, affecting every target organ, including the immune system [195,196]. Macrophages are also considered critical players of obesity-associated immune dysregulation [197,198]. Calpains are involved in the lipid uptake process in macrophages due to the increased activity of the calcium-sensing receptor (CaSR) by extracellular calcium ions in adipose tissue, leading to the generation of a pro-inflammatory adipose tissue environment [199,200]. Moreover, CAPN1 (gene for calpain-1) KO has protected high-fat-diet (HFD)-fed mice from developing liver inflammation, as indicated by decreased levels of oxidized low-density lipoprotein (oxLDL), malondialdehyde (MDA), TNF-α, and IL-6 [201]. Even in apolipoprotein E (ApoE) KO mice subjected to HFD-induced obesity, calpain-1 is critical for inflammatory liver damage [202]. Thus, local and systemic macrophages infiltrate adipose tissue and other organs, such as the liver and kidneys, supporting a pro-inflammatory environment via calpain activation and the overactivation of calpain-dependent pro-inflammatory signaling events, which generate pro-inflammatory cytokines and molecules [203,204]. Moreover, obesity is a critical risk factor for inflammaging, and specific calpain inhibition has protected kidneys from inflammaging via various anti-inflammatory mechanisms, including reduced production of pro-inflammatory molecules and cytokines from macrophages related to NF-κB and NLRP3 inflammasome activation [205]. Hence, calpain targeting in macrophages may exert anti-inflammatory action during acute and chronic inflammatory conditions, depending on the disease type and organ-specificity of the macrophage. However, organ and tissue-specific heterogeneity in macrophages must be kept in mind while exploring the calpain-dependent inflammatory and immunoregulatory functions of macrophages [156].

#### 2.3.2. Neutrophils

Neutrophils are the most abundant (50–70%) leukocytes in human blood. They are the first innate immune cells to migrate to the site of infection and inflammation, where they counteract external or endogenous inflammatory threats. The constitutive calpain expression and activity in resting neutrophils negatively regulate their protrusion and migration, and specific calpain-1 inhibition induces neutrophil polarization and chemokinesis [206,207]. Furthermore, aging neutrophils in homeostatic or in vitro culture conditions undergo apoptosis due to calpain-dependent rearrangements in the protein composition and structure of the plasmalemmal cytoskeleton, such as the dissociation of proteins from F-actin and the loss of α-actin and ezrin proteins, two actin-binding, membrane-anchoring proteins (Figure 3B) [208]. This constitutive apoptosis of aging neutrophils occurs due to a synergism between cytosolic calpains and the proteasome, which is downstream of caspases and critical for limiting inflammation by supporting inflammation resolution, as macrophages clear these apoptotic neutrophils via a process called efferocytosis [208,209]. Failure to resolve the inflammatory phase can induce a chronic stage of inflammation or acute tissue/organ damage, as seen in patients with sepsis [210,211,212]. Thus, calpains are critical for neutrophil homeostasis, and defective calpain signaling may imbalance neutrophil-mediated immune homeostasis or immune regulation, including the death and clearance of senescent neutrophils and their clearance via efferocytosis.

On the other hand, specific knockout of *Capns*1 in MIC, including neutrophils, decreased their infiltration and antimicrobial function at the site of acute bacterial peritonitis [90]. Thus, under homeostasis, calpains serve as negative regulators of neutrophil chemokinesis; however, during infection, inflammation, or in the presence of IL-8 and formyl-Met-Leu-Phe (fMLP), calpains are required for their chemotaxis and migration to the site [90,206]. Moreover, during infection, an increase in neutrophil cytosolic Ca^2+^ activates calpains for their cell spreading (this process is critical for their transendothelial migration and chemotaxis) and phagocytic activity, including phagocytic uptake and ROS-dependent intracellular killing of phagocytosed pathogens (Figure 3B) [90,213,214]. Calpain activation weakens the nuclear envelope by degrading nesprin-1 (an outer nuclear membrane protein) [215]. Additionally, it induces nuclear condensation in response to increased cytosolic Ca^2+^-mediated neutrophil extracellular trap (NET) formation or NETosis, triggered by peptidyl arginine deiminase-4 (PAD4)-mediated histone citrullination (Figure 3B) [215,216,217]. Furthermore, shear stress induces NETosis, which further increases in response to NETosis-inducing agents, such as ATP and lipopolysaccharide (LPS), by activating mechanosensitive ion channel Piezo1 [218]. Piezo1 activation induces calpain activity, which remodels the cytoskeletal architecture, leading to NETosis (Figure 3B) [218]. NETosis is a critical indicator of inflammation severity and also mediates the resolution of inflammation, thereby maintaining immune homeostasis [217,219]. Therefore, calpain inhibition during inflammatory conditions suppresses the inflammatory tissue damage resulting from exaggerated neutrophil infiltration and their inflammatory functions, such as ROS generation, NETosis, and other inflammatory molecules [220]. Hence, studying the impact of calpains on neutrophil functions and behavior is critical for a better understanding of inflammatory and immunoregulatory processes.

#### 2.3.3. DCs

DCs are potent antigen-presenting cells (APCs) that regulate innate and adaptive immune responses under diverse immunological conditions, including tolerance, infections, and inflammatory diseases, such as autoimmunity and cancer [147,221,222,223]. DCs also express the calpain system, which regulates their motility by cleaving actin filaments, the Wiskott–Aldrich Syndrome protein (WASP), β2 integrins, talin, paxillin, and vinculin in their podosomes, thereby controlling the composition and turnover of these structures [224]. Ca^2+^ signaling is critical for DC maturation; however, its effect on cytosolic calpains in this process remains to be explored [225].

Calpain inhibition in primary murine DCs inhibits their transendothelial migration, or diapedesis, to enter lymph nodes and activate antigen-specific T and B cells, which can delay the generation of a protective immune response against infections and vaccines (Figure 3C) [224,226,227,228]. Thus, calpains are critical for DC diapedesis (Figure 3C). Moreover, the presence of the protein tyrosine phosphatase nor receptor type 22 (*PTPN22*)-encoded Lyp phosphatase (Lyp620W) variant increases the risk of autoimmunity by promoting the calpain-mediated cleavage of Lyp/Pep (Pep is the human PTP ortholog in mice) (PTPN22), decreasing Lyp/Pep levels in hyperresponsive lymphocytes and DCs during a steady state [229,230]. It is well established that PTPN22 single nucleotide polymorphisms (SNPs) are associated with several autoimmune diseases, including rheumatoid arthritis (RA), type 1 diabetes mellitus (T1DM), and systemic lupus erythematosus (SLE) [231]. Furthermore, the Janus kinase 2/calpain pathway, in response to platelet-activating factor (PAF), activates PTPIB in monocyte-derived DCs (moDCs), which negatively regulates IL-6 production [232]. As PAF and PTP1B exert immunoregulatory actions on different immune cells, the PAF–calpain–PTP1B axis must be explored to understand immune homeostasis and its dysregulation during inflammatory and infectious diseases [233,234,235]. The immunosuppressive plasmacytoid DCs (pDCs) in human lung cancer TME secrete mature IL-1α due to the activation of absent in melanoma-2 (AIM-2)-like receptor (ALR)-based inflammasomes, which increase intracellular Ca^2+^, activating cytosolic calpains to generate mature IL-1α [236]. Thus, calpains regulate the transendothelial migration and immunoregulatory actions of DCs during inflammatory and autoimmune conditions, which warrants further exploration in response to different PRR and antigen stimuli.

#### 2.3.4. Mast Cells

Mast cells are critical immunoregulatory innate immune cells, primarily known for their role in allergic diseases, such as allergic asthma and dermatitis, as well as anaphylaxis. However, with advances in immunology, their immunological territory has expanded to include infectious diseases, cancers, neurodegeneration, and autoimmunity [145,237,238,239,240,241,242]. The mast cell calpain system is a critical player in immunoglobulin E (IgE)-mediated allergic immune responses, as its inhibition blocks IgE-mediated mast cell degranulation and NF-κB-dependent production of pro-inflammatory cytokines and attenuates IgE-mediated late-phase cutaneous anaphylaxis (Figure 3D) [243]. Activation of mast cells and their calpains in response to glia maturation factor (GMF), along with microglia activation in the methyl-4-phenyl-1,2,3,6-tetrahydropyridine (MPTP)-induced mouse model of Parkinson’s disease (PD), promotes dopaminergic neuron degeneration and progression of the disease (Figure 3D) [244]. Calpain inhibition in the MPTP-induced mouse model of PD has been shown to protect against inflammatory dopaminergic neuronal loss and improve behavioral outcomes [245]. In addition, mast cells are also critical in TBI-induced neuroinflammation, and calpains have been shown to play a role in this process; therefore, it would be interesting to explore the immune cell-specific (including mast cell) roles of calpains in neuro-inflammatory processes [246,247,248,249]. Moreover, calpains are also involved in mast cell adhesion and migration; therefore, their targeting may represent a novel therapeutic approach to control overactive mast cells in different inflammatory diseases [250].

## 3. Calpains in Innate Lymphoid Cells (ILCs)

Phenotypically, ILCs appear as lymphoid cells but do express specific antigen (Ag) receptors, such as T cell receptor (TCR) and B cell receptor (BCR) of T and B cells [251]. There are three major types of ILCs: (1) group 1 ILCs, which include natural killer (NK) cells and ILC1s, secrete IFN-γ and exhibit cytotoxic action; (2) group 2 ILCs, which include ILC2s, secrete type 2 cytokines; and (3) group 3 ILCs, which include lymphoid tissue inducer (LTi) cells and ILC3s, produce IL-22 and IL-17 [251]. The details of ILCs, their role in immunity and infectious and inflammatory diseases, along with their interaction with other immune cells, such as adaptive immune cells, are discussed elsewhere [251,252,253,254,255]. Notably, only NK cells exhibit cytotoxic action; therefore, ILC1s, ILC2s, and ILC3s are considered helper-like ILCs [256]. Studies have indicated the presence of the calpain system in lymphoid cells; however, except for NK cells, their expression in other ILCs has not been studied yet [257,258]. The calpains of activated NK cells play a critical role in the apoptosis of target cells [258]. On the other hand, calpain inhibition in activated human NK cells enhances their cytotoxic action by preventing calpain-dependent protein kinase C (PKC) proteolysis [259]. Interestingly, increased Ca^2+^ influx enhances NK-mediated cytotoxic action, which may regulate early granzyme (Gzm) and perforin, as well as later death receptor-mediated killing of cancer and virus-infected cells [260,261,262]. However, data are limited regarding calpain expression and function in determining the role of ILCs in inflammation and immunoregulation, which requires further exploration.

## 4. Calpains in T Cells

**Figure 4:**
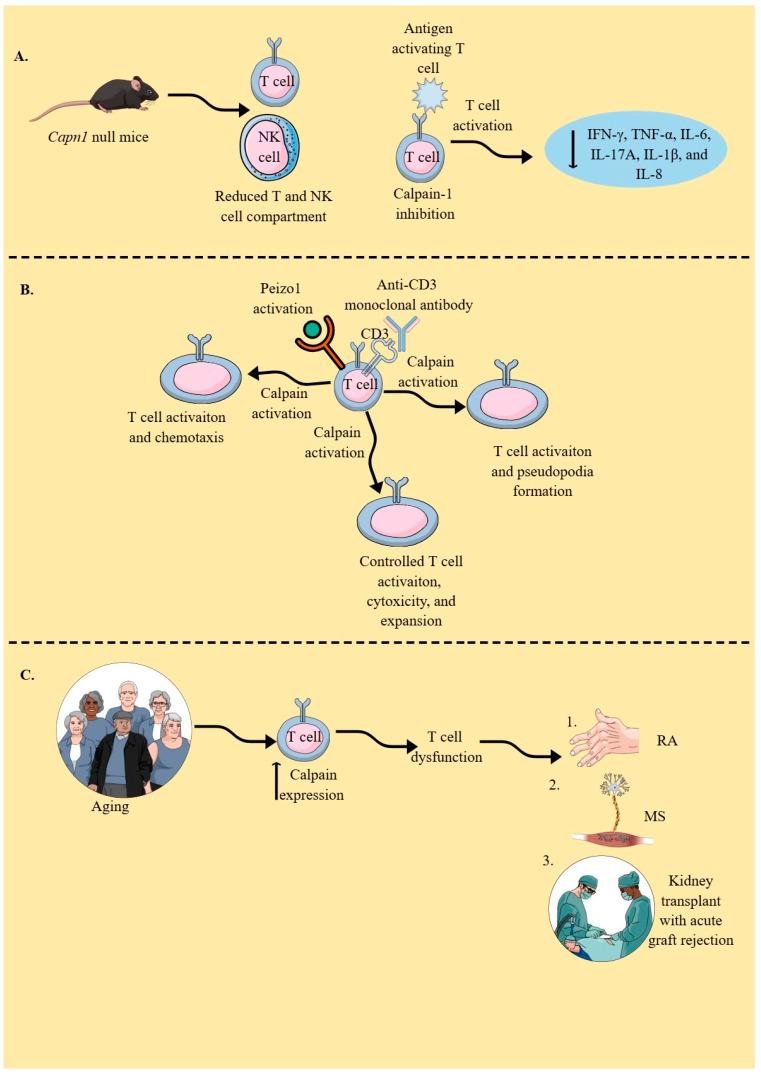
Calpains in T cells. (**A**). Calpain-1 null mice have reduced NK and T cell compartments. Moreover, T cells lacking calpain-1 upon activation secrete lower levels of IFNs and pro-inflammatory cytokines. (**B**). The T cell activation response to anti-CD3 monoclonal antibody activates calpains, which is critical for their pseudopodia formation and mobility. Furthermore, calpain activation regulates their expansion and cytotoxicity. T cell peizo1 activation also regulates their chemotaxis and activation via calpain activity. (**C**). The T cells of older people overexpress calpains, which may induce their dysfunction. For example, T cells isolated from patients with RA overexpress calpains, and T cells infiltrated in acute graft rejection in patients undergoing kidney transplantation also overexpress calpains. Details are mentioned in the text.

T cells, including helper, regulatory, and cytotoxic T cells, play a critical role in maintaining immune homeostasis, and their dysfunction is associated with several autoimmune diseases and cancers [263,264,265,266]. Calpain-1 null mice exhibit consistent expansion in their splenic white pulp (lymphoid hyperplasia) due to an increase in the number and size of follicles and periarteriolar lymphatic sheets (PALSs) [267]. Lymphoid hyperplasia with marked plasmacytosis is also observed in the lymph nodes of calpain-1 null mice, and these mice exhibit multi-organ lymphoid infiltration. T cell and NK cell compartments are reduced in *Capn*1 (calpain-1) null mice (Figure 4A) [267]. The expression of the calpain system in naïve T cells, including several T cell lines, and its upregulation upon T cell stimulation, including anti-CD3 stimulation, has been reported [257,268]. Anti-CD3 monoclonal antibody-mediated activation of T cells activates the calpain system, which cleaves α-actinin, a critical component of actin cytoskeletal assembly and pseudopod formation in activated T cells (Figure 4B) [269].

Even resting CD4^+^ and CD8^+^T cells constitutively express the calpain system, and active calpastatin monitors calpains to avoid their excessive proliferation and the release of various cytokines [270]. For example, calpain-1 inhibition results in the inhibition of various cytokines in stimulated peripheral blood monocytes (PBMCs), including IFN-γ, TNF-α, IL-6, IL-17A, IL-1β, and IL-8 (Figure 4A) [270]. The stimulation of T cells with ionomycin or their adherence to fibronectin activates calpains, which cleave PTP1B to generate active PTP1B, thereby serving as an intracellular checkpoint that limits their expansion and cytotoxicity [271,272]. α4β1 or α5β1 integrin binds to fibronectin in appropriately stimulated T cells, also activating their calpains. The accumulation of calpain-2 and PTP1B at sites of focal contact formation has been reported [273]. The inhibition of calpain in T cells impairs their ability to adhere to and spread on immobilized fibronectin.

Moreover, activation of calpains in T cells upon antigen stimulation degrades TCR-associated zeta chain-associated protein kinase-70 (ZAP-70), which occurs in parallel with TCR internalization and degradation, indicating that calpain activation might control their overactivation, such as overexpansion and cytotoxicity (Figure 4B) [274]. For example, the negative regulation of ZAP-70 serves as a mechanistic basis for the differential expression of CD4^+^ and CD8^+^T cells in thymic and mature T cells [275]. Mechanosensor peizo1 also plays a critical role in T cell activation, which, upon activation, induces Ca^2+^ influx and calpain activation, thereby further organizing the cortical actin scaffolds required for optimal TCR activation (Figure 4B) [276]. Moreover, this peizo1 and calpain axis also regulates T cell chemotaxis (Figure 4B) by inducing an integrin called lymphocyte function-associated antigen 1 (LFA-1) at the leading edge of chemotactic human T cells [277]. Thus, calpain-mediated talin cleavage is critical for LFA-1 activation. However, calpain-4 (*Capns1*), the regulatory component of calpain-1 and calpain-2, is not essential for LFA-1-mediated adhesion, conjugation, or migration of CD4^+^T cells under normal conditions [278].

The strong LFA-1-dependent adhesiveness of T_regs_ to DCs is partly dependent on their lower calpain activities [279]. T_reg_ adhesion to DCs sequesters Fascin-1 (FSCN-1), an actin-bundling protein (critical for immunological synapse formation), and skews Fascin-1-dependent actin polarization in DCs toward the T_reg_ adhesion zone [279]. This T_reg_-DC interaction, or immune synapse, which is MHC-II independent, induces lethargy in DCs, rendering them unable to induce the potent T cell priming that supports the T_reg_-mediated stage of immunosuppression or immunoregulation [279,280]. Talin1 is critical for TCR-induced adhesion of T cells to ICAM-1 and T cell-antigen-presenting cell (APC) conjugation or immune synapse formation [281]. However, cleavage of talin by calpains is critical for focal adhesion disassembly. It serves as a rate-limiting step during adhesion turnover, as this process also affects the disassembly of other adhesion components, including paxillin, vinculin, and zyxin [282]. For example, the talin–vinculin axis is a key mechanosensing component of cellular focal adhesions, as vinculin molecules bundle actin and localize to focal adhesions in a force-independent manner, requiring talin [283,284]. Thus, calpains are critical regulators of T cell activation, proliferation, and migration/chemotaxis in response to various routes of stimulation, which warrants further study due to the emergence of contradictory findings.

The expression of calpain in T cells increases with aging, indicating its role in T cell (CD4^+^ and CD8^+^) dysfunction, such as proliferation and associated pro-inflammatory functions (Figure 4C) [285]. For example, the cell cycle of CD4^+^T cells in the healthy elderly population is significantly shorter than that in the young population due to the overexpression of cyclin D, which is attributed to their lowered degradation by calpains [286]. Thus, it will be interesting to investigate calpain expression in T cells isolated from healthy elderly individuals and those with various diseases, such as cancer, neurodegenerative diseases, and metabolic syndrome, to explore their specific roles in regulating T cell-mediated immunity (TCMI) in the aging population. T cell stimulation also increases calpain secretion through their *ATP-binding cassette transporter* (ABCA1) transporters, which may modify the inflammatory immune environment [268,287]. For example, extracellular calpain activates anti-inflammatory TGF-β, inactivates pro-inflammatory chimerins, decreases IL-17 expression in murine Th17 cells by inducing the shedding of TLR2, helps in the regeneration of injured epithelium, and supports angiogenesis [287,288,289,290,291,292,293].

The calpain activity in T cells isolated from patients with rheumatoid arthritis (RA) is higher than that in healthy controls (Figure 4C) [294]. Moreover, RA patients develop calpastatin autoantibodies, and their incidence is higher than in other patients with systemic autoimmune diseases, such as systemic lupus erythematosus (SLE, 27%), polymyositis/dermatomyositis (24%), systemic sclerosis (38%), and overlap syndrome (29%) [295,296]. The inhibition of calpains with a membrane-permeable cysteine protease inhibitor (E-64-d) in experimental arthritis models served as a therapeutic targeting. Thus, the calpain–calpastatin system may play a critical role in inflammatory cascades associated with autoimmune diseases. For example, the calpastatin–calpain balance is disturbed during Th1, Th2, and Th17 development, as calpastatin (CAST) overexpression or calpain inhibition with E-64-d suppresses IL-6 and IL-17 production by Th cells and IL-6 production by fibroblasts, due to reduced RORγt expression and STAT3 phosphorylation [297]. Patients undergoing kidney transplants with acute graft rejection exhibit overexpression and activation of calpain-1 in their infiltrating T cells (Figure 4C) [298]. Even calpain inhibition, achieved by silencing its small regulatory subunit (*Capns*1), inhibits Th17 development. Conversely, calpastatin overexpression inhibits IL-17 production from Th17 cells by overactivating STAT-5 signaling. Thus, the calpain system must be studied during helper T (Th) cell polarization, as it is critical to immune responses during diverse inflammatory conditions, such as cancer, autoimmunity, and infectious diseases.

Calpain inhibition in mice with skin transplants increased the duration of skin allograft survival and diminished T cell infiltration into the allograft. It is interesting to note that calpastatin overexpression in T cells decreased calpain levels and their migration but increased their proliferation due to amplified IL-2 signaling via stabilization of the IL-2R common γ-chain [298]. Thus, calpain inhibition delays allograft rejection by decreasing T cell infiltration but not proliferation. Moreover, increased calpain activity is associated with increased levels of pro-inflammatory Th1 cells in patients with multiple sclerosis (MS) (Figure 4C), and calpain inhibitor treatment of PBMCs isolated from patients with MS decreases T cell proliferation, elevates their indoleamine 2,3-dioxygenase (IDO) levels, and downregulates Th1/Th17 inflammatory cytokines [299,300]. Moreover, IDO is a critical immunosuppressive molecule that catabolizes cytosolic tryptophan to induce immunosuppressive effects, including the induction of T_regs_ [301,302]. Thus, T cell-specific calpain inhibition can decrease the pro- and auto-inflammatory functions of T cells during MS. Hence, calpains govern the immunoregulatory and pro-inflammatory functions of T cells, including their polarization into different Th phenotypes, which must be further explored to understand their roles in various T cell-mediated inflammatory conditions, such as GVHD, autoimmunity, and cancers.

## 5. Calpains in B Cells

Ca^2+^ signaling in response to B cell receptor (BCR), PRR, cytokine, and chemokine signaling is critical for survival, proliferation, differentiation, and migration of B cells to lymphoid and target organs; therefore, its disruption may induce cell death or abnormal proliferation, as seen in different leukemias [303,304]. Calpains are Ca^2+^-dependent cytosolic cysteine proteases that are also expressed in B cells, and their expression increases in CD19^+^B cells of aging individuals [285]. *Capn*1 null mice exhibit an overall increase in the number of B cell lineage cells in the spleen and bone marrow, along with an increased myeloid-specific GR1^+^, CD11b^+^ granulocytic cell lineage and megakaryocytic CD41^+^ cells [267]. Notably, pancreatic islet B cells only express calpain-1 [305]. Calpain activation downstream of BCR signaling is critical for B cell clonal deletion, a process that eliminates autoreactive B cells, and the establishment of the B cell repertoire, which occurs due to the activation of caspase 7 (CASP7). In contrast, overexpression of its inhibitor (calpastatin or CAST) inhibits BCR-induced apoptosis in immunoglobulin M-positive (IgM^+^) cells [306,307]. Moreover, CD40 ligation during BCR signaling downregulates calpastatin levels, which support calpain activation in controlling B cell clonal deletion and establishing the B cell repertoire [307]. Activated calpains also cleave the Myc protein, which stimulates B cell differentiation and amplifies Ca^2+^ signaling, and its dysregulation may lead to various B cell malignancies [308,309,310]. Myc is also critical for the formation and maintenance of the germinal center (GC), and c-Myc^+^GC subpopulations of B cells may increase the risk of malignant transformation [311]. Hence, the calpain–Myc axis in GCs and B cell malignancies must be explored.

Overactive calpain-1 in chronic B cell leukemia (B-CLL) and childhood acute leukemia blasts (ALL-Bs) has been reported to further increase with age and prevent their apoptosis [294,312]. However, there is hope in the form of calpain inhibitors. Treating these leukemia B cells with calpain inhibitors increases their apoptosis in a dose-dependent manner, offering a potential avenue for treatment. The increased calpain activity in B-CLL is associated with decreased activity of pro-apoptotic caspases, such as CASP3 and CASP9, and increased activity of anti-apoptotic Bcl-2 protein [313,314]. Moreover, calpain inhibition (calpain inhibitor II or CPI-2) also induces apoptosis among acute lymphoid leukemia (ALL) and non-Hodgkin’s lymphoma B cells, which is dependent on caspase activation but not on the protein tyrosine kinases LYN or Bruton’s tyrosine kinase (BTK) [315].

Furthermore, calpain-1 overexpression correlates well with phosphorylated zeta-associated protein 70 (pZAP-70) in B-CLL lymphocytes. Interestingly, the proportion of CD19^+^ B cells with overexpressed and active calpain-1 and pZap-70 significantly decreases in patients with B-CLL after successful therapy [313]. TLR2 activation in B cells during *S. flexneri* infection induces their apoptosis. Therefore, it is crucial to investigate calpain activation, as TLR2 activation induces calpain activation in different innate immune cells, as discussed earlier [316,317]. Understanding calpain activity downstream of BCR and PRR signaling in B cells under diverse infectious and inflammatory conditions, such as cancers and autoimmunity, is of utmost importance. This knowledge can help us develop more effective vaccine and immunotherapy candidates to target infectious diseases and cancers, underscoring the urgency and significance of our research.

## 6. Future Perspectives and Conclusions

The controlled working of the immune system (immune cells and their humoral factors) is critical to maintain tissue/organ homeostasis and immune homeostasis [318,319]. The dysregulated immune response in response to infections, allergens, injuries, and disturbed homeostasis due to several endogenous factors, such as DAMPs, generates an inflammatory milieu, which, if not resolved or treated, proves detrimental to the host through tissue or organ damage, leading to organ loss or function, auto-inflammatory or autoimmune disease induction, and cancer development [12,320]. The calpain system is present in all innate (except ILCs, as no data are available) and adaptive immune cells and regulates their death, function, and migration/mobility to or away from the site of infection/inflammation. For example, downstream immune cell activation mechanisms, in response to several MAMPs, PAMPs, and DAMPs via cytosolic Ca^2+^ disturbance, impact calpain activity, affecting immune regulation and inflammatory processes [321]. Moreover, calpains are also critical to BCR and TCR downstream signaling, essential for the antigen-dependent immune response of adaptive immune cells (B and T cells).

Controlled calpain activity is crucial for immune cell migration across both epithelial and endothelial barriers, suggesting that their epithelial and endothelial cell-specific targeting has the potential to treat inflammatory diseases. For example, different calpain-specific therapeutic strategies have been developed with better bioavailability and specificity of calpain inhibitors to target various inflammatory diseases, such as cancers, calpainopathies, neurodegenerative diseases, TBI, and several other inflammatory diseases [322,323,324,325,326,327,328]. Although calpain targeting is emerging as a novel pharmacotherapy for several inflammatory diseases, including cancers, data regarding immune cell-specific targeting are scarce. Therefore, understanding immune cell functions in the context of the calpain system may be a helpful approach to further understand inflammatory and immunoregulatory processes critical for host defense and pathogenesis of different inflammatory diseases. Furthermore, these studies will provide us with an opportunity to develop immune cell-specific calpain targeting with disease specificity. For example, DC-specific calpain activation during infectious diseases and cancers, where DC migration to LNs helps generate an antigen-specific immune response, such as effector T and B cells, will provide us with the opportunity to develop potent adjuvants to increase the efficacy of currently available vaccines and therapies. Even antimicrobial peptides (AMPs), such as cathelicidin LL-37, induce apoptosis in target cells, including cancer cells and infected cells, by activating calpain along with the apoptosis-inducing factor (AIF) pathway [329,330]. Thus, AMP-mediated calpain upregulation can be utilized to combat intracellular infections and cancers in an immune cell-specific manner [331,332].

The selective deletion of *Capn*1 and *Capn*2 in endothelial cells reduces cardiac fibrosis and hypertrophy while also alleviating myocardial dysfunction by supporting angiogenesis and preventing apoptosis in mice with diabetes, a metabolic syndrome condition characterized by elevated chronic systemic inflammation [333]. Moreover, calpain inhibition in pancreatic islet cells increases insulin release by enhancing exocytosis of insulin granules [334]. Thus, calpain inhibition may be protective in metabolic syndrome associated with diabetes; therefore, it would be interesting to determine how it affects individual immune cells responsible for the systemic chronic inflammation seen in these patients.

In conclusion, the immune cell-specific calpain system (calpain-1 and calpain-2) is an area that is starting to gain recognition. However, further exploration is crucial to fully understand and develop immune cell-specific targeting of this system to control inflammatory diseases and associated immune dysregulation. The functional regulation of every immune cell, like that of any other cell, is governed and affected by the cytosolic Ca^2+^ flux, a critical regulator of calpain. Therefore, calpains must be studied in the context of specific immune cell function and regulation. They not only cleave cytoskeletal proteins but are also associated with NLRP3 inflammasome activation, IL-1β, adhesion molecule expression, antigen presentation, and the cleavage of several other non-cytoskeletal proteins. Hence, a calpain lens is crucial for understanding inflammation and immune dysregulation to re-establish immune homeostasis. The potential for further exploration and the development of immune cell-specific targeting strategies make this an exciting area for future research.

## Data Availability

Not applicable.
